# Placental Presentation

**Published:** 1859-10

**Authors:** Wm. H. Baltzell

**Affiliations:** Chicago


THE CHICAGO

### MEDICAL JOURNAL.

VOL. II. OCTOBER, 1859. No. 10.

### ORIGINAL COMMUNICATIONS.

ARTICLE I.

### PLACENTAL PRESENTATION.

BY WM. H. BALTZELL, M.D., OF CHICAGO.

Very early in the morning of Monday, July 5th, 1858, I was summoned to attend upon Mrs. M——, in labor with her fifth child. Her husband, who was the messenger, informed me on our way to the house, that his wife on the Friday night previous had been taken with the first symptoms of labor, indicated by occasional fugitive pains, which were accompanied with slight discharges of blood from the vagina; that the pains had gradually increased in frequency and severity, and that the hemorrhage had become profuse and alarming. He also informed me that he had secured the services of a midwife, who, among other absurdities, had told him that "there was no child," that "the bleeding proceeded from a diseased growth in the womb," etc. Upon arriving at the house, I found the woman in a condition such as to excite my most serious apprehensions; her features were pinched, contracted, and perfectly bloodless; her whole body was bathed in a clammy sweat; her voice was scarcely above a whisper, and upon the slightest attempt at exertion, a condition approaching syncope supervened.

I immediately made an examination, and found the os uteri yielding and dilatable, with, as I had anticipated, the placenta presenting; the waters had been discharged about five hours before, without however diminishing the flow of blood. It was a case of central placental presentation, and upon introducing my hand and passing the finger between it and the uterus, I found that it was detached completely upon the right side of the cervix, but adherent upon the left; the size of the seat of its attachment being, as far as I could judge, rather more than one-third of its uterine superficies.

Impressed with the necessity of prompt interference, if I would save the life of the mother, who indeed already had the appearance of a person moribund, and conceiving the child's death more than probable, and at all events its state secondary to the mother's safety, I separated the remaining attachments of the placenta, and withdrawing my hand was gratified to observe the almost immediate cessation of the hemorrhage.

A few minutes after I had detached the placenta, a pain occurred, discharging it into the vagina; after removing it from this situation, I could readily detect the head of the child presenting in the second position of the vertex. I then administered ergot, more with a view to its effects in insuring the contraction of the uterus after it might be emptied of its contents, than with a desire of its specific action upon that organ in the expulsion of the child, for, notwithstanding the alarming debility of the woman, the uterine contractions had been moderately powerful all along. There was no more hemorrhage of any moment, and a few pains completed the birth of the head. I however delayed somewhat the delivery of the body, for fear of the effect of too rapidly emptying the uterine cavity in the excessive prostrate condition of my patient. The child, as I had expected, was dead; it was well formed, and considerably above the average size.

The uterus contracted very well, and nothing untoward occurred. The woman was enjoined against making any exertion whatever, was placed upon a nutritious diet, and allowed a moderate quantity of stimulants; and though for a long time feeble and anemic from her great loss of blood, yet in about three months she had completely regained her usual health and robust appearance.

With regard to the amount of blood lost, I can, of course, form only a proximate estimate. The apartment in which the woman was confined had very much the appearance of a butcher's shambles; she was lying in a large pool of coagulated blood, which had also soaked through two mattresses and half filled a chamber vessel which had been placed to receive it, besides, in a corner of the room there was a large and confused mass of sheets, towels, etc., some merely stained, others completely saturated with blood. I believe that I do not exaggerate when I place the quantity lost altogether, from Friday night until the delivery of the child, at considerably upwards of sixty ounces.

It is very much to be regretted that in cases of placenta prævia, confessedly one of the most dangerous and fatal complications that can occur in labor, there should be so many various and conflicting opinions among medical men as to the proper method of procedure. This is due, in some measure, to the obscurity involving the exact relations existing between the placental and the maternal circulation; and still more from a natural tendency to generalize individual experience, and inaugurate theories from data, obviously, in many instances, insufficient. Hence, it may not be uninteresting cursorily to examine some of the various methods advised by different obstetrical writers, with a view of discovering, if possible, which mode is most clearly based upon received physiological principles, and which, in its application, is best adapted to secure the safety of the mother and child, or if they be incompatible, the safety of the mother.

First, as to the operation of perforating the placental mass, either with the fingers or a pointed instrument, and so introducing the hand through the opening to perform version by the feet. Besides the difficulty of rending the tissue in question, Dr. Meigs, in his excellent work, has clearly and succinctly exposed the fallacy and *uselessness* of this proceeding. It renders the child liable to death from the rupture of the blood vessels of the placenta, and the consequent hemorrhage; it is apt to retard the delivery of the head by its being obliged to pass through that mass; and it may occasion the very difficulty, to avoid which its advocates recommended the operation, viz., the detachment of the placenta in the process of delivering. There are other objections unnecessary to specify, as, notwithstanding its approval by many writers, and, I believe, the teachings of some of the Philadelphia schools, the practice has nearly gone out of vogue; it merely resolves itself into turning with the superaddition of unnecessary difficulties. Next, as to the expedient of turning, strenuously recommended and established as an obstetrical maxim by almost all the writers and teachers of the present time, and most generally practised by physicians at this day. We are told that we must carefully introduce the hand, if possible, at the point at which the placenta is detached, and cautiously passing it between the walls of the uterus and the membranes, rupture them high up, and seizing the feet of the child, perform the version.

Notwithstanding the unanimity of standard authorities in obstetrical science upon this topic, and notwithstanding its recommendation upon what certainly seem to be correct principles, viz., the emptying of the womb, so as to permit the condensation of its substance, and the obliteration of the vessels furnishing the material for the hemorrhage,—still, the indiscriminate and exclusive employment of this measure, as a rule of practice, when artificial assistance is indispensable, can be shown to be attended with a fearful maternal mortality greatly dependant upon the operation itself, and in very many cases avoidable. It will be understood that in the use of the terms "indiscriminate" and "exclusive," no reference is had to the injudicious or uncalled for interference of ignorant or inexperienced practitioners, for to do this would prevent the elucidation of a correct principle, but deductions have been made from the statistical tables of physicians of enlarged experience, admitted judgment, who have made obstetrics their special study. As has been ably shown by Dr. Simpson, the limited experience of any one or a few persons, in the investigation of the worth of an operation, is an uncertain guide in instructing us as to its general and determinate value; reliable conclusions can only be obtained by the critical examination *in the mass* of the results in a large number of cases. When this test was applied to the practice of turning in placenta prævia, and the immense ratio of mortality attendant upon it was shown, to a certain extent, the medical world was taken by surprise.

The statistical information was mainly procured from the reports of lying-in hospitals under the charge of accomplished accoucheurs, where the danger could not be supposed to be increased by the accidents of ignorance, prejudice or officiousness on the part of the medical attendant.

Out of 421 cases collated by Simpson, "in which the child was removed by turning, the result was fatal to the mother in 144 cases; or, in other words, the mothers were lost in the proportion of more than 1 in 3 (1 in 2.9). Upon the continent of Europe, the Cæsarian operation, reputed the severest in midwifery, has been fatal, according to Dr. Churchill, in 154 out of 371 cases, or 1 in 2.4" (Simp., 1st series. p. 718).

This exhibit, the correctness of which cannot be disputed, clearly shows the frightful fatality attendant upon turning as a *rule* of practice, in the accident of labor under consideration; and it certainly merits reflection before we reject a measure *which can be proved* greatly to supersede the necessity of an operation, the danger of which differs from that of the Cæsarian section by but .4, and is more than twice as great as in lithotomy. And is it surprising that the proportion of deaths should be so heavy?

Dr. Lee has characterized turning under the most favorable auspices as a most painful and dangerous proceeding; and it is easy to imagine how the peril is increased when the recuperative powers of the system are broken down by vast loss of blood, and the woman is terror-stricken at her rapidly failing strength, and unnerved and agitated by the alarm of her attendants. These considerations, if they be correct, conclusively demonstrate the great risk involved in the indiscriminate resort to this measure, even in cases where active assistance is imperatively demanded; and we are brought to the investigation of the plans, by means of which it is claimed that the danger, to a great extent, is diminished. As is of course generally known, the advice of Dr. Simpson consists in the complete detachment of the placenta previous to the delivery of the child. He was induced to favor and advise this course from a consideration of exceeding loss of life in turning, and from the satisfactory termination in several cases that came to his knowledge, in which spontaneous expulsion of the placenta was followed by cessation of hemorrhage. Having adopted the expedient of removing it, in some desperate cases that happened in his own practice, with a like result, he was led into an inquiry as to the value of the remedy as compared with turning, deriving his information from statistical reports. Of 141 cases, in which the placenta was delivered previous to the child, there were 10 maternal deaths, or 1 in 14, and a number of these could reasonably be attributed to other causes than a loss of blood. This striking difference between the two modes of relief conclusively show the force and benefit of his discovery as affecting the safety of the mother in comparison with turning.

It is not the intention of this paper to enter into the particulars of Dr. Simpson's theory; this can be done profitably by any one by perusing his elegant essay. But the *fact* seems incontrovertible, that the complete separation of the placenta in unavoidable hemorrhage from the presentation of that body, is an efficient, comparatively safe, and generally successful means of restraining the flooding. It has been seen that in my case the detachment was almost immediately followed by a stoppage of the flow, and I believe it justifiable and advisable in all instances of great exhaustion and danger from the continuance of the bleeding. Whilst, however, adopting the practice of Dr. Simpson, it is not, I think, essential for us to receive, as correct and conclusive, *all* the principles laid down by him as explaining its propriety and success.

In this connection it may be stated, that the effect upon the child is by no means so necessarily fatal as has been asserted by the opponents of the practice, which is readily susceptible of proof. Out of the 141 cases of premature expulsion of the placenta, there are 106 given in which the result as affecting the child had been noted; of these, 73 were born dead and 33 living, or, 31 per cent. were saved and 69 per cent. were lost (Simp., 1st series, p. 621).

Out of 70 cases reported by the two Ramsbothams (appendix), a similar account was kept in 40, and the number of the children saved was 8, or only 20 per cent., 80 per cent. having been lost.

As having some bearing upon this subject, it would be well to recollect that in pelvic presentations generally, under ordinary circumstances, the mortality to the child is about 1 in every 5 (Meigs, p. 398). But it is not pretended that placental detachment is an operation for the benefit of the fœtus. As to the causes of the cessation of the hemorrhage, it can, I think, be in a great measure explained, without adopting the opinion of Dr. Simpson concerning the entrance of the mother's blood into the substance of the placenta, and its flowing into the uterine cavity from the open vessels of that body. It is a mooted question whether the maternal blood enters any portion of the placenta at all; many writers, as Meigs, Velpeau, Ramsbotham, etc., taking the opposite view; and even the great majority of those who suppose that it does, agree in asserting that in unavoidable hemorrhage, the blood is lost directly from the uterus. If, as Carpenter affirms, the functions of the placenta—that is, the æration, and, perhaps, other changes in the fœtal blood—are performed in the substance of that viscus itself, at the junction of the so-called maternal and fœtal portions, and the villous expansions of the umbilical vessels are situated about that locality, it must be a positive condition, as necessary for the interchange of elements and the life and development of the child, that in all cases the maternal blood should so circulate.

There can be no more exception to this rule conditional with life to the child, than there can be exceptional instances of oxygenation of the blood without the necessity of air entering the lungs. In other words, if it be admitted that the expanded extremities of the bloodvessels of the cord are the instruments for the performance of exosmosis and endosmosis, and if they be situated in the middle of the placenta, the mother's blood must enter the placenta, or there will be no nutrition afforded the child; and if, on the other hand, it can be indubitably proved, not in one but in many cases, that the bloodvessels of the mother have carried that fluid no further than the inner surface of the womb, and that nevertheless the fœtus developed, grew and lived, it is *more* than presumptive evidence against the placento-maternal circulation. For instances of this kind, reference can be made to Velpeau, p. 206; Meigs, pp. 207 and 208; a very interesting case reported by Dr. Henry Madge, which forcibly illustrates this view (Braith., part 33, p. 259).

Independently of this question, the spongy structure of the placenta itself is unfavorable for the rapid transmission of blood, and it is lost in large gushes in placenta prævia. Further, in a case of inversio uteri, Dr. Merriman *saw* the blood issuing from the surface of the womb (Braith., part 36, p. 217). Finally, it is *unnecessary*, in explaining the cessation of hemorrhage, to adopt that portion of Dr. Simpson's theory referring to its immediate source.

In consulting the works of nearly every author upon this subject, one is struck with the unanimity with which they advise expedients, all proposed with regard to a mechanical effect alone; overlooking, in a great measure, the fact, that the uterus, although invested with special and peculiar functions, is as much under the control of and subject to the general physiological laws of the system as any other organ of the body. Dr. Simpson has adverted to this subject, and shown the inconsistency of the omission; and it is his reflections upon this point that seem worthy of adoption, as satisfactorily solving the problem in dispute, when taken in connection with, though often sufficient alone and independent of, the mechanical effect of the pressure of the child and the contraction of the uterine fibre. There is, undoubtedly, an "attractive power" resident in the placenta when affixed to the uterus, which determines an increased quantity of maternal blood to that organ, for the necessary purposes of the nutrition of the fœtus and the essential changes in its blood. This stimulus continues, probably in a proportionate degree, so long as any portion of the placenta is attached to the womb; but when the "normal irritation" of its adherence ceases, the extra supply of blood, sent there for a special purpose, is diverted to the general circulation, and under these circumstances, slight mechanical action is generally sufficient to control the flow. In the same manner, under the stimulus of the presence of food, the blood is directed in large quantities to the coats of the stomach, its capillaries are distended and receive an unusual supply, but after the process of digestion is completed, the surplus of blood requisite for this functional office falls again under the organic laws of the circulation of the general system.

Examples of this kind might be multiplied, but however this physiological principle may have been overlooked in its relation to the subject under discussion, its bearing upon it can hardly be denied. So long, therefore, as any portion of the placenta adheres to the womb, there is an increased determination of blood to that point; and as the vascular sinuses of the uterus in that position communicate with each other, and are supplied with blood from the same maternal vessels, a large quantity must necessarily escape from the patulous orifices left by its partial separation.

If these premises be correct, therefore, without adopting the circuitous placento-maternal circulation believed by Dr. Simpson, we may, in a great measure, explain the *fact* of there being a greater hemorrhage from partial than complete detachment of the placenta, and why the latter, as a remedial operation, has a great tendency to restrain the flooding, and is *most generally* efficient to do so. The practice of the tampon in rigid os uteri, and the expedient of drawing off the liquor amnii, have not been dwelt upon, because they are tentative measures, and whilst as such proper and advisable in many cases, still are very frequently only preliminary to the choice between turning and detachment, though it may be remarked that the evacuation of the waters adds greatly to the danger and difficulty of the former method.

Let us now briefly examine "The New Physiology of Placenta Prævia," by Dr. Robert Barnes and Dr. Cohen, of Hamburgh. They advocate the separation of the placenta from the cervix or dilating portion of the uterus only, or, in their language, "the conversion of a central into a lateral placenta." There seem to be several inconsistencies in and objections to this practice. It is believed by them that the hemorrhage proceeds directly from the surface of the womb, and not from the placenta; and quoting from Sir Charles Bell, relative to the peculiar arrangement of the muscular fibres of the uterus, it is stated by him in the very article selected that it is the dilating portion or cervix that supplies the hemorrhage, the surface of which they propose further to increase, leaving the placenta attached only to the contracting part of the womb. That the flow proceeds from the dilating cervix ("which relaxation is something quite different from a mere yielding to pressure, and is obviously a vital phenomenon that marks a peculiarity in the actions of this part,"—Carp., p. 979), is proved by the fact that the hemorrhage is greater during a pain; whilst the body of the womb contracts, the cervix yields and dilates, of course allowing the blood to pass more freely and in larger quantities. From the very structure of the placenta itself, it is very probable that in all cases of placental presentation it is quickly detached from the dilating walls. The maternal face is divided into a number of lobes or placentulæ, with deep sulci or furrows between them. When these are applied to the concave surface of the womb, the corresponding side of the placenta is necessarily convex, and, of course, its superficies is greater than if it were a plane. The furrows become more and more narrow, according to the degree of contraction of the womb, until at length becoming obliterated, further condensation of the muscular tissue throws off the placenta. By placing one of these bodies into a concave glass, as one of the shades for ornamental clocks, a plain view can be had of its uterine face, and it will be seen that the sulci are arranged to admit of considerable contraction of the womb, without impairing the attachment of the placenta. We are all familiar with breech cases, in which, after the waters had passed, the body of the child delivered, and the womb contracted to half its size, the pulsation of the cord continued, indicating the persisting connection between the womb and placenta.

On the other hand, in prævial cases, how slight the dilatation necessary to cause alarming flooding, many cases being reported in which it occurred when the os could hardly be felt to be dilated at all, showing the attachment of the placenta to be such as readily to admit of its being broken by this relaxation, and the degree required to be very moderate.

If this be so, as the labor advances it would seem unavoidable for the separation to occur in the cervix. Besides, the practice of this plan of Dr. Barnes has not been sufficiently tested to clearly recommend its utility, and certainly *seems* founded upon incorrect principles.

